# Epidermolysis Bullosa in Spain: A Qualitative Analysis of Its Social Impact on Families With Diagnosed Minors

**DOI:** 10.1111/hex.14128

**Published:** 2024-06-29

**Authors:** Juan Manuel Martínez‐Ripoll, Marta García‐Domingo, Yolanda María de la Fuente Robles

**Affiliations:** ^1^ Family Support Department DEBRA Spain Málaga Spain; ^2^ Department of Psychology, Faculty of Social Work University of Jaén Jaén España

**Keywords:** Epidermolysis Bullosa, family, impact, reality

## Abstract

**Background:**

Epidermolysis bullosa (EB) comprises a group of rare types of genodermatoses characterized by extreme mucocutaneous fragility, leading to blistering and/or erosions, even with minimal trauma. Continuous care through wound management is an integral part of daily life for the families and individuals affected. The aim of this study was to assess the social reality and impacts on families of having minor members diagnosed with EB in Spain.

**Methods:**

A qualitative methodology was employed, utilizing four focus groups entailing participation by 24 parents (19 mothers and five fathers) of minors diagnosed with EB in Spain.

**Results:**

Negative impacts on the family nucleus were evident in four priority areas of analysis: sociorelational, economic‐labour, physical and psychoemotional, with significant differences observed based on the severity of the symptoms.

**Conclusion:**

Impacts on the family nucleus are noticeable from birth, influencing all other daily life routines and complicating family planning and organization. There is an imperative need to enhance the availability of sociohealth resources and to adopt an interdisciplinary approach to address their biopsychosocial needs.

**Patient or Public Contribution:**

The active participation of relatives of minors diagnosed with Epidermolysis Bullosa (EB) is invaluable to sociohealth professionals, legislators and researchers. A team member conducts their professional activities at DEBRA España (national patient association dedicated to enhancing the quality of life for individuals with EB and their families), actively engaging in all study phases.

## 1 Introduction

### 1.1 Epidermolysis Bullosa (EB): Types, Epidemiology and Complications

EB is a heterogeneous group of rare forms of genodermatosis characterized by extreme mucocutaneous fragility, leading to blistering and/or erosions, even with minimal trauma [1]. To date, more than 30 subtypes of EB, varying in severity, have been described and classified based on the severity of blisters and/or erosions. Its diagnosis, therefore, is complex, and its classification has undergone recurrent revisions. According to the latest consensus classification, EB can be grouped into four types: EB simplex, dystrophic EB, junctional EB, and kindler EB [1].

Due to its low prevalence worldwide, obtaining precise and updated quantification data is challenging. In the latest data published its calculated prevalence rate was 11.1 per million inhabitants, with an incidence of 19.6 per million live births [1]. All types of EB are associated with skin and/or mucosal fragility, though to varying extents. Clinical manifestations can range from blisters and/or erosions on the hands and feet to severe extracutaneous complications [2]. Among these, squamous cell carcinoma stands out as the leading cause of mortality [3].

Given the absence of any cure for EB, the sociohealth strategies established aim to alleviate symptoms through wound care, bandaging, and delaying potential complications [1]. Thus, the complexity of EB is evident, not only for the individual diagnosed but also for the family nucleus.

### 1.2 The Impact of EB on the Family Nucleus: Quantitative and Qualitative Evidence

Due to its chronic and incurable nature, the disease's impacts on the family nucleus are felt from the first day of the patient's life [4]. Individuals with EB require meticulous and continuous care, resulting in physical and mental deterioration for primary caregivers and affecting the quality of life and emotional well‐being of the family nucleus [3].

Numerous studies, both qualitative and quantitative, have analyzed the disease's social reality and its impact on the family nucleus across different cultural contexts, yielding similar results regardless of publication date. All studies point to a significant emotional impact, acknowledging feelings of life dissatisfaction [5], guilt, sadness [6], fear of loss, suffering, and helplessness [7, 8].

Furthermore, inequality and discrimination in accessing sociohealth resources due to a lack of information and awareness pose an additional challenge for families [9, 10], with the economic and labour impact being highly noticeable [11]. Increased family expenses are one of the most recurrent problems [12], compromising social relationships and even marriages [11, 13, 14].

This economic impact is exacerbated by factors such as the need to reduce or abandon paid employment. In this regard, mothers shoulder a greater impact due to their role as primary caregivers [8, 13, 14], acknowledging feelings of guilt for neglecting other healthy children [15].

Research conducted thus far provides relevant information about the social reality of families with a minor diagnosed with EB and, consequently, its impact on the family nucleus. However, it is crucial to generate higher levels of evidence from a sociorelational and familial perspective to comprehensively understand and address the challenges these families face. Our current study contributes to achieving this goal.

### 1.3 Current Study

Existing studies have provided valuable quantitative and qualitative evidence, indicating significant emotional and economic repercussions on families with minors diagnosed with EB. However, to obtain a more complete and detailed representation of the social reality, it is imperative to address the complexities of family relationships and access to sociohealth resources.

Therefore, the main objective of this research was to assess the social reality and impacts on the family nucleus where a minor diagnosed with EB exists in Spain. Specifically, inquiries were made regarding family dynamics and the sociohealth resources available based on the population of residence and type of EB. All of this, with the aim of addressing our initial research question: *What is the impact on the family dynamics of Spanish households with a child diagnosed with EB?*

The analysis of the collected data aims to generate knowledge about the social reality of families with minors diagnosed with EB in Spain.

## 2 Methods

### 2.1 Research Design

The hermeneutic phenomenological approach was employed to investigate the experiences of the participants with the purpose of developing and understanding how individuals attribute meaning to these experiences [16].

This qualitative methodology employed the focus group (FG) technique, allowing for exploration of the perceptions and subjectivities of the participating families. The FG technique was deemed the most suitable for this study because it is able to capture the contradictions, nuances, and arguments of the participating families. Therefore, the analysis of the data collected aims to generate knowledge of the social realities of families with minors diagnosed with EB in Spain.

### 2.2 Participants, Procedure and Instrument

The study involved 24 families (19 mothers and five fathers) of minors with EB, after obtaining their informed consent and ensuring confidentiality and anonymity. Table 1 summarizes the participating population.

**Table 1:** Sample sociodemographics (*N* = 24 participants).

Demographics	*N*	%
Relationship with the diagnosed minor		
Father	5	21
Mother	19	79
Employment status		
Employed	12	50
Unemployed	12	50
Marital status		
Married	21	87
Divorced	3	13
Type of EB		
EBS	10	42
JEB	1	4
DDEB	1	4
RDEB	12	50
Nationality		
Spanish	24	100
Residence population		
+20,000 inhabitants	12	50
−20,000 inhabitants	12	50

Abbreviations: DDEB, dominant dystrophic epidermolysis bullosa; EB, epidermolysis bullosa; EBS, epidermolysis bullosa simplex; JEB, junctional epidermolysis bullosa; RDEB, recessive dystrophic epidermolysis bullosa.

The participating families were recruited through collaboration with DEBRA España (a national patient association aimed at improving the quality of life for people with EB and their families). Intentional sampling was used by sending participation invitations to families residing in Spain who met the established criteria. A total of 36 families were contacted, out of which 24 agreed to participate.

To ensure a precise and representative analysis, capable of extracting similarities and differences in the heterogeneous reality of the different population groups studied, parents with children diagnosed with different types of EB and residing in disparate locations participated. Within EB types, those with mild symptoms were considered for EB Simplex localized (EBS), Junctional EB (JEB), and Dominant Dystrophic EB (DDEB) with localized involvement, while those with severe symptoms were considered for Recessive Dystrophic Epidermolysis Bullosa (RDEB). Regarding the Spanish resident population, a ±20,000 inhabitants' criterion was used as a differentiating point. The differential point is set at ±20,000 inhabitants to assess the influence of the division of competences within the Spanish public administration on access to sociohealthcare resources, as well as the potential differential access to specific support resources depending on the typology of the reference municipality. These inclusion criteria enable the control of confounding factors associated with the severity of EB, the age of the minor, and the demographic characteristics of the population, thereby contributing to minimizing bias and reinforcing the internal validity of the study.

Based on these criteria, four FGs were organized:

- Parents of minors with RDEB residing in cities with more than 20,000 inhabitants in Spain.
- Parents of minors with EBS, JEB, or DDEB residing in cities with more than 20,000 inhabitants in Spain.
- Parents of minors with RDEB residing in cities with less than 20,000 inhabitants in Spain.
- Parents of minors with EBS, JEB, or DDEB residing in cities with less than 20,000 inhabitants in Spain.

For script development and the identification of topics to be addressed, internationally validated scales were used as references: Epidermolysis Bullosa Burden of Disease EB‐BoD [17] and the Family Dermatology Life Quality Index [18]. Previous studies on the topic were also consulted for discussion purposes.

A customized script was developed with a total of 32 questions distributed across six areas, including leisure and social well‐being, sociohealth resources, occupational environment, economic situation, primary caregiver overload, and continuity of care. An example of a FG question is ‘What aspects of EB do you consider most exhausting?’

Due to the geographical dispersion of the families, the FGs were conducted online, with an average duration of 90 min, between March 2023 and May 2023.

### 2.3 Data Analysis

The FG sessions were recorded, transcribed, and analyzed using the ATLAS.ti qualitative analysis software. The content established received priority, although spontaneous themes or contradictions in the discourse were also analyzed in this study.

**Figure 1:**
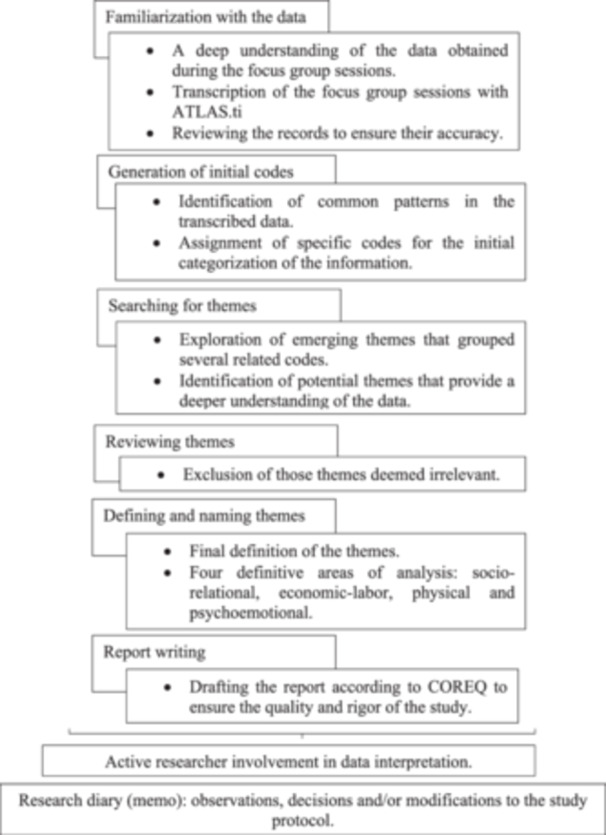
Summary of thematic analysis. COREQ, Consolidated Criteria for Reporting Qualitative Research.

The collected data underwent a rigorous reflexive thematic analysis in six phases, following the methodology proposed by Braun and Clarke [19]. Initially, we immersed ourselves in a deep understanding of the data obtained during the FG sessions. Then, after transcribing the sessions, reviews were conducted to ensure the accuracy of the records. Common patterns were identified and assigned codes for data categorization. Subsequently, these were refined and reviewed, excluding any that were deemed irrelevant. Finally, after a detailed content analysis, the results were structured into four areas of analysis: sociorelational, economic‐labour, physical, and psychoemotional. Figure 1 summarizes this data analysis.

The guidelines established by the Consolidated Criteria for Reporting Qualitative Research (COREQ) were considered to ensure the quality and rigour of the study [20]. As outlined by Braun and Clarke [19], the reflexive approach aimed to acknowledge the active involvement of the researcher in interpreting the data. In this regard, researchers were mindful of their personal reflection through a research diary (memo), where observations, decisions, and/or modifications to the research protocol were recorded, aiming to enhance the reliability of the process [20, 21].

The input was anonymized and coded to ensure the confidentiality required, as committed to by the researchers through the informed consent document. The codes correspond to father (♂) and mother (♀), followed by the EB type (EBS, JEB, DDEB, and RDEB), the age of the minor (N°) and the residence population, with a larger population (P+) and smaller population (P−). These elements were separated by a forward slash and followed by a number in parentheses, corresponding to the numbering assigned to each participant.

## 3 Results

### 3.1 Reality and Impact on Families of Minors With Mild Symptoms (Localized EBS, JEB and DDEB)

**Figure 2:**
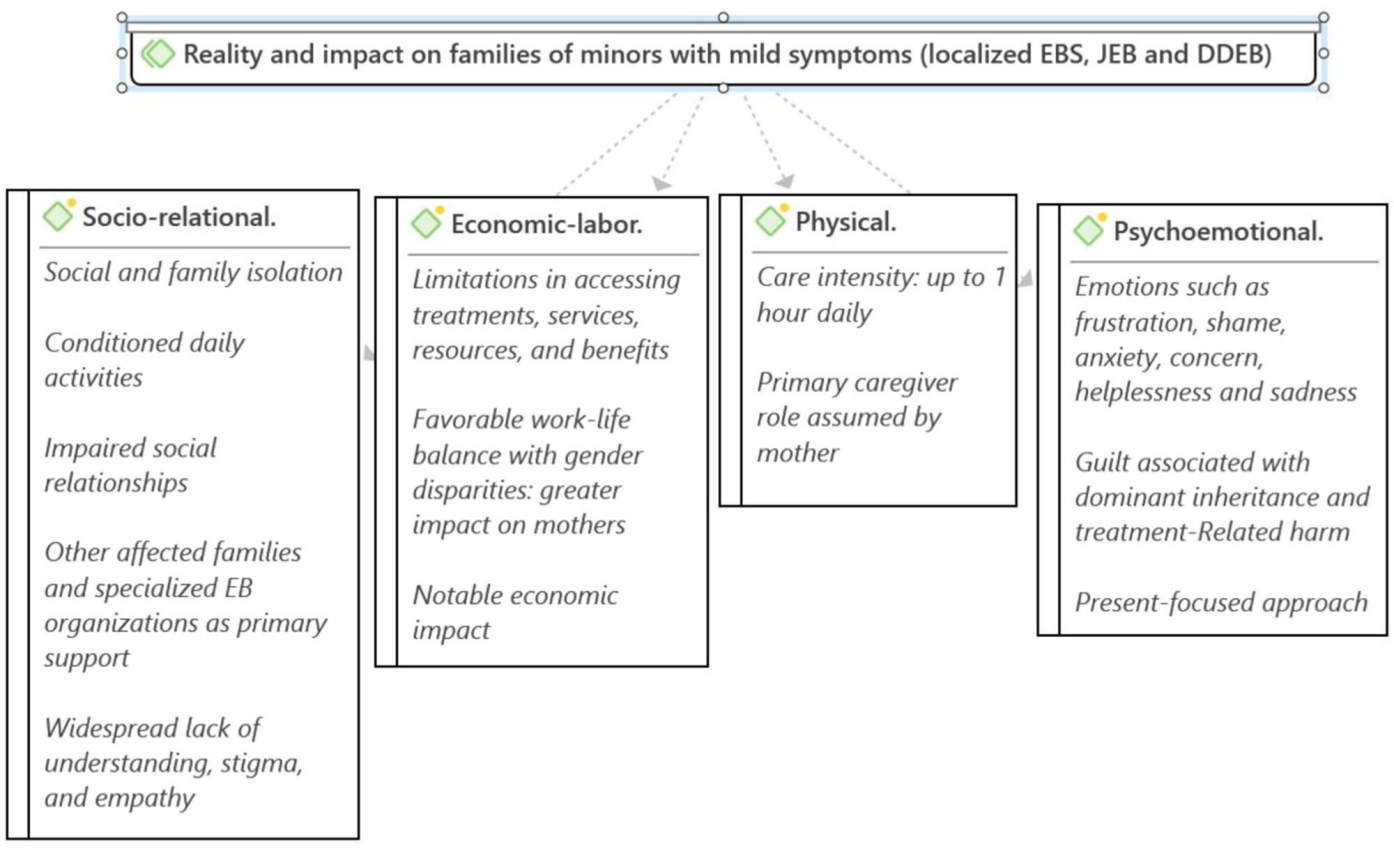
Reality and impact on families of minors with mild symptoms (EBS, JEB and DDEB). DDEB, dominant dystrophic epidermolysis bullosa; EBS, epidermolysis bullosa simplex; JEB, junctional epidermolysis bullosa.

The participating families shared a wide variety of situations, circumstances, and emotions experienced within their family environments, and it was possible to identify and categorize this input into different areas of analysis: sociorelational, economic‐labour, physical and psychoemotional. Figure 2 summarizes the reality shared by participating families according to the areas of analysis established.

#### 3.1.1 Sociorelational

At the intrafamily level, the dynamics of families with children diagnosed with EB exhibiting mild symptoms do not undergo significant changes. Despite adversities, marital relationships do not appear to deteriorate significantly in this group, thanks to the potential for a well‐organized family structure.

However, concerning leisure activities and vacation periods, families are constrained by the need to avoid exposure to heat and long‐distance outdoor ambulation, critical aspects for the management of EB symptoms. This constraint not only impacted the families' recreational activities, but also led to social isolation within the family units.

We engage in activities with family and friends. We try to avoid walking long distances, and heat.

♀/EBS/15/P+ (14)

When we travel, we prefer to go alone because we can't keep up with other families.

♀/EBS/3/P− (16)

We also prefer to travel alone, so we can go at our own pace.

♀/EBS/15/P+ (21)

At the extrafamily level, social relationships have been impaired by the constraints and limitations imposed by complications associated with EB.

The pace of life and concerns are not the same. Our friends have left us, although I have also distanced myself from them.

♀/JEB/9/P+ (13)

In this context, support from other families facing similar situations, as well as organizations like DEBRA, and specialized EB centres, is considered crucial for the well‐being of the family unit.

We feel supported by DEBRA; thanks to them, we met more families that make you feel understood.

♀/EBS/8/P− (17)

However, a recurring lack of understanding, stigma, and widespread empathy deficits toward EB appear in society, negatively affecting families' quality of life and emotional well‐being.

At the pool, at the supermarket, at the beach… I've been asked if it's contagious or if she got burned.

♀/EBS/8/P− (17)

The doctor said he would notify the social worker, thinking that I caused my daughter's injuries. There is a lack of empathy in the healthcare field.

♀/EBS/14/P+ (21)

Families of adolescents affected reported perceiving feelings of shame and misunderstanding in their minors when they are seen in public, negatively impacting their social skills and adversely affecting the family's social participation. They expressed feeling more understood in their own municipalities than outside them.

My son is embarrassed to be seen at the beach. He pays more attention to the looks he gets.

♀/EBS/16/P− (19)

#### 3.1.2 Economic/Labour

The lack of knowledge and awareness of EB poses a constant challenge when it comes to accessing treatments, services, resources, and benefits. Families reported not receiving any benefits and even feel reluctant to reapply due to previous negative experiences. This sense of exclusion made them feel detached from the reality of EB, highlighting the urgent need for greater awareness and the promotion of knowledge.

We don't receive any help, for disability or dependence. I refuse to apply again.

♀/EBS/15/P+ (14)

Most of these families have been able to combine caring for their child with the work of both parents, although some mothers have had to leave their jobs or reduce their working hours since the child's birth, leading to serious financial and labour repercussions.

We have always both worked, and we manage with the help of family.

♂/EBS/8/P+ (11)

The purchase of the products and treatments necessary to manage EB entails economic sacrifices for some families.

They supply us with everything at the health center. Initially, I had to pay and then request reimbursement. Currently, we have to buy some moisturizer.

♀/EBS/15/P− (18)

#### 3.1.3 Physical

The intensity of daily care can amount to up to an hour per day. Although they strive to balance task distribution between both parents, mothers typically take on most of the care, generating an added emotional and physical impact. However, families did not notice that this time dedicated to care influenced their personal time or their ability to make decisions.

Now that he's older, he takes care of himself. When he was little, it took about 45 minutes to do the care.

♀/EBS/15/P+ (14)

#### 3.1.4 Psychoemotional

Families suffer from seeing their children unable to engage in the same activities as others. As they grow, the first questions arise, and the first feelings of life dissatisfaction emerge, significantly impacting family members.

He can't do all the activities his friends do. If he plays soccer one day, he knows he won't leave the house for four days. Seeing him suffer is difficult.

♀/EBS/15/P− (18)

They verbalized that EB has been a taboo topic at home and acknowledged feeling guilty for harming their children during care and even for passing on the disease to the child through dominant inheritance. These feelings of guilt affected the emotional health of the family nucleus and their relationships with the disease.

It also hurts me when I do the care, as if someone were stabbing my heart. Besides, I notice that my husband feels guilty about his dominant inheritance.

♀/EBS/7/P+ (15)

Families with minors at early ages expressed concern about EB development, sharing feelings of anxiety and unease.

It's quite localized and, for now, we're keeping a close eye to see how it evolves and how he grows.

♂/EBDD/1/P+ (12)

However, families with teenagers report greater peace of mind due to their children's adaptation to self‐care, alleviating the primary caregiver's burden.

They adapt and know what they have to do, and if they do it, they know what comes next; and tomorrow will be another day.

♀/EBS/12/P− (20)

Families prefer to live in the present rather than worry about future situations, helping to reduce anxiety and stress related to EB.

### 3.2 Reality and Impact on Families of Minors With Severe Symptoms (RDEB)

**Figure 3:**
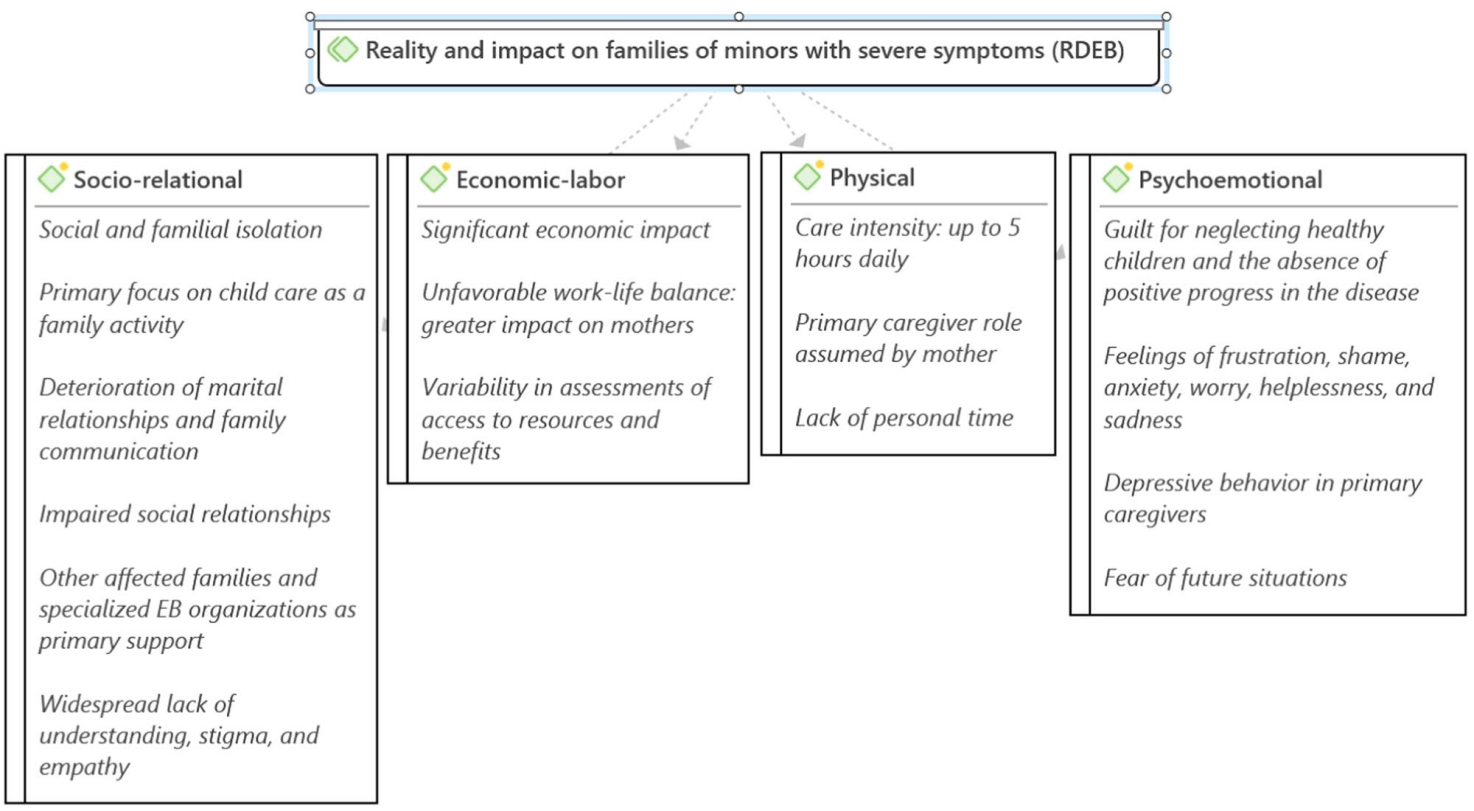
Reality and impact on families of minors with severe symptoms (RDEB). RDEB, Recessive Dystrophic Epidermolysis bullosa.

Similar to the previous group, the shared reality of families allowed for categorization into different areas of analysis: sociorelational, economic‐labour, physical and psychoemotional. Figure 3 provides a synthesis of this group.

#### 3.2.1 Sociorelational

At the intrafamily level, the dynamics of families with children diagnosed with severe EB symptoms are negatively impacted due to the demands of care. The prioritization of essential activities, such as treatments and attendance at various therapies (physical therapy, speech therapy, early intervention, etc.) significantly affects the normal activities of the family unit and limits the parents' ability to devote time to other activities, with these families expressing the need for external and professional help for caregiving.

Between the treatments, school, and therapies, she hasn't had time to be a child.

♀/EBDR/5/P+ (3)

The most entertaining thing my son does is watch cartoons during the treatment.

♀/EBDR/3/P− (24)

We need a professional to come home to evaluate our child occasionally because we don't have the knowledge of a nurse.

♂/EBDR/6/P− (10)

Attention to the needs of the child impacts family communication and marital relationships, affecting their quality of life and decision‐making capacity. These feelings were intensified by the negative impact on work/life balance.

The couple's relationship is affected by the caregiving burden and the scarcity of time for each other. Some couples mentioned the difficulty of finding moments to be together and the emotional exhaustion this entails, which, in some cases, has led to separations due to differences in coping with the disease.

I separated from my husband because we experienced the illness differently. I haven't found a job compatible with caring for my daughter.

♀/EBDR/12/P− (9)

At the extrafamily level, similar to the previous group, support from other families and organizations, like DEBRA, and specialized EB centres, is crucial for the well‐being of the family unit.

Regarding the families' relationships with formal support resources, they expressed a lack of empathy and widespread misunderstanding, especially in the healthcare field, where professionals have sometimes actually worsened the child's condition due to mishandling.

In the first vaccine, the nurse thought he knew what he was doing, applied an adhesive bandage, and removed it. We went home with a new wound.

♂/EBDR/6/P− (10)

#### 3.2.2 Economic/Labour

Access to resources and benefits, due to the visible impact of the child's condition, is not an obstacle, although they highlighted variability in assessments due to a lack of knowledge. This underscores the importance of greater awareness of and education about EB.

The heavy burden of caring for the child makes it impossible to balance care with the work of both parents, leading to economic benefit applications, reduced working hours, or complete job abandonment. Similar to the previous group, the impact on employment was more pronounced in mothers.

I returned to work, and it was impossible. Eventually, I quit my job.

♀/EBDR/6/P− (6)

My wife has requested a 50% reduction.

♂/EBDR/10/P− (7)

At first, we both worked. Now I prefer to reduce my hours by 99%. I'm at her disposal, and she is more at ease.

♀/EBDR/13/P− (8)

My wife stopped working.

♂/EBDR/6/P− (10)

They emphasized the economic effort required to access specialized EB services in their community and, mostly, outside it, resulting in a monthly cost that can be triple that of the previous group. Access to specialized services is crucial for the child's care and well‐being.

#### 3.2.3 Physical

The intensity of care for these children significantly increases, requiring 4–5 h daily. This creates an imbalance in the distribution of roles, as, despite attempts to provide care support, it is primarily administered by mothers, who acknowledge having taken on the role of the primary caregiver.

I prefer to do the treatment myself because, if I don't participate, I feel worse.

♀/EBDR/13/P− (8)

My wife does the treatments because she prefers it that way.

♂/EBDR/10/P− (7)

I give the bath, and my wife does the treatment.

♂/EBDR/6/P− (10)

If it weren't for my wife, I don't know how I would do it. Doing the treatments alone is very difficult.

♂/EBDR/5/P+ (5)

Lack of time for themselves is a common issue for these families. Constant attention to the needs of the child with EB makes it challenging to find time for self‐care and personal or couple activities. The family reports not having time for themselves and mentions feeling overwhelmed, exhausted, and mentally fatigued, negatively impacting their emotional well‐being and caregiving capacity.

We try to have separate spaces because together is complicated.

♀/EBDR/6/P− (6)

I don't feel like doing anything because of how he suffers and what hurts him.

♀/EBDR/12/P− (9)

#### 3.2.4 Psychoemotional

When the children show no improvement in the disease's treatment, parents often feel frustrated and guilty about the lack of progress.

You think you're not doing something right, but it is what it is.

♂/EBDR/5/P+ (5)

Feelings of helplessness and constant fear when engaging in family or age‐appropriate activities for the child are common emotions. They report feeling alone and isolated, displaying expressions of discouragement and depressive attitudes.

We're afraid to do certain activities, and, if something happens, the logistics are tremendous. This disease consumes you, and you can't leave it. We feel the need to breathe occasionally.

♂/EBDR/10/P− (7)

They expressed that their children are not like other children, and require continuous care, and acknowledged feeling guilty for not giving the same attention to their other healthy children.

I have another daughter. We don't spend time with her, and the little we do isn't enough. I feel terrible for her, but I don't have the time.

♂/EBDR/5/P+ (5)

Families described emotions such as frustration, shame, worry, anxiety, and features compatible with depression. They expressed difficulties accepting the disease and the daily burden, along with feelings of helplessness and sadness when seeing their child in pain and suffering.

The good of my daughter is that I have to hurt her.

♀/EBDR/5/P+ (3)

Thinking about future situations elicited feelings of distress and restlessness, accentuated by fears of possible end‐of‐life stages they have experienced or learned about from other individuals affected. Fear of the unknown produces constant concern.

Don't think, live day by day. You have to set a barrier, and whatever happens will happen.

♀/EBDR/11/P+ (2)

I think it's a normal process because we see the reflection of our children. We have to move forward; it's a constant internal struggle.

♀/EBDR/9/P+ (1)

During the treatment, I'm not a mother; I'm a nurse. Then I'm a mother, and I cry.

♀/EBDR/12/P− (9)

When we go to treat her, you take out your heart and treat your daughter. Otherwise, you're not able to do it.

♀/EBDR/5/P+ (3)

Families expressed a strong need for informational support, especially regarding current research and clinical trials, which offer a small but important dose of hope.

## 4 Discussion

The results underscore that irrespective of the type of EB, symptomatology, or residential population, families share similar realities. The complexity of EB is evident, impacting not only the individual diagnosed but also the family nucleus [3], leading to a loss of well‐being and a decline in quality of life.

This study delved into various analytical realms—sociorelational, economic‐labour, physical, and psychoemotional—employing the FG technique, with participation of 24 parents (19 mothers and five fathers). This approach allowed for an exploration of perceptions and subjectivities within families caring for an EB‐diagnosed child in Spain.

Families with minors diagnosed with EBS, JEB, or DEB do not experience significant alterations in their daily lives compared to families with RDEB. However, constraints on certain family activities persist, regardless of the severity. A lack of empathy and understanding is perceived by both groups, leading to conflicts across various domains of care, and even evoking feelings of shame about being seen in public [9, 10]. Nevertheless, families residing in municipalities with populations under 20,000 reported a higher level of empathy compared to those in more populous areas.

Concerning family relationships, a more pronounced deterioration is observed in families with RDEB, as noted in the study by Macik and Kowalska‐Dąbrowska [5]. Likewise, the care and attention given to the child affect social and marital relationships, impacting family communication and decision‐making capacities [11, 13, 14].

The lack of understanding towards families with minors with EBS, JEB, or DEB results in a reduction in access to resources and benefits, a situation not shared by families with RDEB, due to its severity and visibility. In this context, access to resources and benefits is more influenced by subjective evaluations than the residential population.

Similarly, work‐life balance and the caregiving burden vary widely between the two groups, with RDEB families deeming them incompatible. The labour impact is more noticeable in women, who face reductions, abandonment, or losses of job opportunities, as noted in other qualitative research [6, 7, 8].

Likewise, the economic impact is greater among RDEB families, who experience recurrent displacements and/or hospitalizations at specialized EB centres [22]. In contrast to other studies, access to treatment does not impose such a pronounced economic impact due to its free provisioning by the Spanish National Health System.

Physical strain is pronounced among RDEB families, requiring up to 5 h of daily care, with mothers predominantly assuming roles as primary caregivers, significantly impacting personal and family time, according to prior research [8, 13, 14].

Families reported feelings of frustration, shame, anxiety, depression, guilt, loneliness, lack of acceptance, and life dissatisfaction [5]. Families of minors with RDEB exhibited more pronounced depressive behaviours than those in the other group. Fear of future situations (improvement or deterioration) encourages both groups to hold onto a glimmer of hope for future research and/or clinical trials, with the findings of Gowran et al. [9] and Kearney et al. [10].

Prior research focuses on families of minors with RDEB, overshadowing families of minors with localized mild conditions. However, this study demonstrates that families of minors with EBS, JEB, and DEB also suffer a negative impact on their family dynamics.

Given the existing impacts on the family nucleus, irrespective of the type of EB and residential population, the need for sociohealth resources to increase awareness and understanding of EB becomes apparent.

### 4.1 Limitations

Among the study's limitations was the difficulty of guiding families to speak broadly rather than focusing primarily on the child's situation, considering EB as a disease that dictates the daily life of the afflicted. Families showed reluctance to discuss the disease's impact on the parents, preferring to focus on the child's experience with EB. Bearing in mind the rarity of the disease, another limitation was the number of participants. Despite inviting both parents, the participant population was predominantly composed of women, limiting insights into the experiences perceived by men within the family nucleus. This highlights a disparity in the distribution of caregiving responsibilities based on gender, a phenomenon common in Spain as well as other parts of the world, the thorough exploration of which in future research could enrich the understanding of family dynamics and contribute to improving available evidence. Lastly, population variables were considered to examine potential disparities in available sociohealth resources; however, no significant differences were identified in this regard.

### 4.2 Implications

The findings contribute to a clearer understanding of the reality faced by families with children diagnosed with EB. There is an imperative need for greater awareness and education both within the general society and among healthcare professionals. This measure would significantly reduce the stigma associated with the condition and foster a deeper and more empathetic understanding towards families with children diagnosed with EB.

Additionally, given the wide range of negative emotions they experience, providing access to psychological services and creating a supportive environment can greatly enhance their emotional well‐being. Ensuring equitable access to resources and benefits for all families affected by EB is another crucial aspect, especially for those facing economic and caregiving challenges.

Therefore, it is essential that future research focus on implementing specific strategies that address the healthcare, psychological, and social needs in an interdisciplinary manner to improve the care of children with EB and their families. The findings of our study will be beneficial in enabling a comprehensive understanding of the reality faced by families with children diagnosed with EB.

## 5 Conclusions

This study aimed to evaluate the social realities and impacts on the nuclei of families with children diagnosed with EB in Spain. Through a qualitative analysis, it can be concluded that, regardless of the type of EB and the child's residential population, impacts on the family nucleus are noticeable from birth, and are decidedly negative. These impacts range from affecting daily routines and social and marital relationships to producing feelings that result in physical and psychoemotional deterioration for both parents.

## Author Contributions

**Juan Manuel Martínez‐Ripoll:** conceptualization, writing–original draft, methodology, writing–review and editing, formal analysis, data curation, software, resources, investigation. **Marta García‐Domingo:** supervision, formal analysis, writing–review and editing, methodology, validation, investigation. **Yolanda María de la Fuente Robles:** supervision, writing–review and editing.

## Conflicts of Interest

The authors declare no conflicts of interest.

## Data Availability

The data supporting this study's findings are available on request from the corresponding author. The data are not publicly available due to privacy or ethical restrictions.
